# Adherence to the EAT-lancet dietary pattern among older adults in Rwanda and its association with micronutrient intake

**DOI:** 10.29219/fnr.v69.12174

**Published:** 2025-08-18

**Authors:** Theogene Habumugisha, Anna Stubbendorff, Penias Tembo, Eric Matsiko, Inger Elisabeth Måren, Matthias Kaiser, Karin Borgonjen-van den Berg, Alida Melse-Boonstra, Ingunn M. S. Engebretsen, Jutta Dierkes

**Affiliations:** 1 Centre for International Health, Department of Global Public Health and Primary Care, University of Bergen, Bergen, Norway; 2 Nutritional Epidemiology, Department of Clinical Sciences Malmö, Lund University, Malmö, Sweden; 3 Arnold School of Public Health, Department of Epidemiology and Biostatistics, University of South Carolina, Columbia SC, USA; 4 Cancer Prevention and Control Program, University of South Carolina, Columbia SC, USA; 5 Department of Human Nutrition and Dietetics, College of Medicine and Health Sciences, University of Rwanda, Kigali, Rwanda; 6 Department of Biological Sciences, University of Bergen, Bergen, Norway (IM); 7 Centre for the Study of Sciences and Humanities, University of Bergen, Bergen, Norway (MK); 8 Division of Human Nutrition and Health, Wageningen University and Research, Wageningen, The Netherlands; 9 Centre for Nutrition, Department of Clinical Medicine, University of Bergen, Bergen, Norway

**Keywords:** sustainable diet, plant-based diets, nutrients, micronutrients, aging, sub-Saharan Africa

## Abstract

**Background:**

Sub-Saharan Africa is facing a dietary transition with both undernutrition and rising rates of non-communicable diseases. Adopting the reference diet proposed by the EAT-Lancet Commission can reduce both the environmental burden and improve health outcomes. However, whether this diet provides micronutrient adequacy in older adults in low-income settings has not been investigated. This study examines adherence to the EAT-Lancet diet and its association with micronutrient intake among older adults in the Gasabo district, Kigali, Rwanda.

**Methods:**

This cross-sectional study involved 334 older adults aged 55–93 years from Kigali, Rwanda. Dietary intake was assessed using two non-consecutive 24-h recalls, and socio-demographic data were collected through interviews. Adherence to the EAT-Lancet diet was evaluated using a 42-point index based on food group consumption and micronutrient intake was analyzed in relation to adherence tertiles. Multivariable regression models, adjusted for age, sex, and energy intake, were used to assess associations between diet adherence and micronutrient intake.

**Results:**

The EAT-Lancet adherence score ranged from 13 to 36, with a mean of 24. Higher adherence was associated with increased consumption of whole grains, vegetables, fish, and nuts, while red meat, dairy, and poultry intake declined. Adherence was positively associated with energy intake and intake of retinol-equivalents, thiamine, niacin, folate, vitamins B12 and D, calcium, phosphorus, magnesium, iron, and zinc. When adjusting for energy intake, the association with zinc and potassium became non-significant and the association with riboflavin became negative.

**Conclusion:**

Higher adherence to the EAT-Lancet diet was associated with higher intake of energy and several micronutrients important for healthy aging in this population of predominantly older, poor adults in Kigali. However, the potential for nutrient gaps, particularly in riboflavin, highlights the need for context-specific dietary adaptations to ensure nutritional adequacy in older populations in Rwanda.

## Popular scientific summary

- **What’s new:** This study is the first to explore the relationship between adherence to the EAT-Lancet diet and dietary intake in older adults in sub-Saharan Africa, with a focus on Kigali, Rwanda.
- **Implications:** Higher adherence to the EAT-Lancet diet improves micronutrient intake. The findings highlight the dual benefits of environmentally sustainable and healthy diets within low-income settings.

Many dietary patterns of today are harming both human health and environmental sustainability. Globally, the food sector (including production and consumption) accounts for 30% of greenhouse gas (GHG) emissions (1), with a significant portion originating from animal-based foods (2). In absolute terms, low- and middle-income countries (LMICs) have seen the sharpest rise in food-related emissions over recent decades – increasing by 53% in low-income countries and 12% in middle-income countries – compared to a 3% increase in high-income countries (HICs) (3).

In LMICs, food-related environmental burden is driven by urbanization, population growth, and economic development, triggering a dietary shift from traditional plant-based diets toward Western diets, rich in meat and processed foods (4, 5). Sub-Saharan Africa (SSA) is one of the LMIC regions with the fastest-growing population, and its population is expected to quadruple by 2100, reaching 4 billion (6, 7). The largest share of this population growth (80%) is also expected to occur in urban settings (8).

Like in other regions, the older population is also growing in LMICs, and the WHO estimates that over three-quarters of older adults (aged ≥ 60 years) will be living in LIMCs by 2050 (9). This trend presents growing public health challenges, particularly in ensuring adequate nutrition for aging populations. Globally, over 2 billion people are still affected by undernutrition and micronutrient deficiencies (10, 11). SSA is disproportionately affected, and it is the only region where dietary micronutrient density has declined during the last 50 years (10, 12). In SSA, the prevalence of malnutrition is estimated to range from 6 to 54% among older persons (13).

From dietary perspectives, micronutrient deficiencies can be addressed by the consumption of micronutrient-rich foods, such as animal-sourced foods (ASFs), which on the one hand have often higher micronutrient density and bioavailability, but on the other hand these foods have detrimental effects on the environment (14–17). It has been estimated that a high-meat diet has four times the environmental impact of a plant-based diet in terms of GHG emissions and land use, nearly triple the impact on biodiversity, and double the water use (18).

A shift towards more sustainable diets, rich in plant-based foods, might therefore mitigate the environmental impact from food and is a requirement for achieving the United Nations’ Sustainable Development Goals (SDGs) (19, 20). Given these health and environmental challenges, the EAT-Lancet Commission on healthy diets from sustainable food systems introduced the ‘planetary health diet’ in 2019 for minimizing environmental impact, maintaining dietary adequacy and lowering the risk for non-communicable disease (NCD) (16). Being a universal reference diet, the EAT-Lancet diet, emphasizes the intake of plant-based foods such as wholegrains, vegetables, fruits, legumes, nuts, and unsaturated oils, moderate amounts of fish and poultry, and restricts the intake of added sugar, dairy, and red meat. The diet has been shown to reduce climate impact and other environmental damage related to the human diet (21, 22). The diet has also been shown to be associated with positive health outcomes such as lowering the risk of mortality, diabetes, and cardiovascular disease in populations from HICs (21, 23–25).

Although following dietary patterns such as the EAT-Lancet diet has the potential to reduce NCDs and the diet-related environmental footprint, there are also concerns that adopting such dietary patterns would aggravate nutrient deficiencies, including micronutrient deficiencies, in the population groups with higher nutritional requirements, such as the elderly, women of reproductive age, and young children (26, 27). Micronutrients are critical for healthy aging, supporting immune function, cognitive health, bone integrity, and the prevention of (NCDs) (28). Older adults are especially susceptible to micronutrient deficiencies due to physiological changes, diminished dietary intake, burden of chronic diseases, and restricted access to diverse foods.

Despite these concerns, only few studies have examined the EAT-Lancet diet in populations from LMICs (29–32), and the studies focusing on micronutrient adequacy are, however, scarce, especially in older populations with low intake of ASFs. The Rwandan context provides an opportunity to evaluate the nutrient intake of the older population in relation to plant-forward dietary patterns, such as the EAT-Lancet diet, in SSA. The number of the older population is also growing in Rwanda due to rising life expectancy, which increased from 65 years in 2012 to 70 years in 2022 (33). Like many other SSA countries, the dietary patterns of many Rwandans are also predominantly plant-based (34). Therefore, this study aimed to assess the micronutrient intake in relation to adhering to the EAT-Lancet diet among older adults in Kigali, Rwanda.

## Methods

### Study design and context

This cross-sectional study was conducted in the Gasabo district of the City of Kigali in Rwanda, during November 2021 and January 2022. This study was conducted as a part of a larger project investigating the impact of diets on healthy aging in SSA. The project focused on understanding the implications of replacing (or lacking) ASFs on the dietary intake and body composition of the older population. This goal aligns with recommendations such as the EAT-Lancet diet, which advocates for minimal to moderate consumption of ASFs (16). To achieve this, the project employed qualitative and quantitative (dietary survey) study designs to obtain insight into both quantitative measures of the diet and consumer perspectives (35, 36).

### Study population

This study analyzed the data from the participants who were enrolled in the larger project. In the larger project, the sample size was computed based on the estimated difference in protein intake (9 g/d, standard deviation [SD]: 36) between the older adults consuming plant-based diets and those consuming diets rich in ASFs (37). Considering alpha = 0.05 and power = 80%, the estimated sample size was 376 older adults, and the final targeted sample size was estimated to be 414 participants when considering a 10% non-response rate.

The participants were recruited from the Gasabo district through a two-stage cluster random sampling process. In the first stage, villages were randomly chosen using a list of villages and in the second stage, households with older adults from each village were randomly selected using a list of households compiled by the research team in collaboration with community health workers in the selected villages. If more than one person per household was eligible, only one older adult was randomly chosen to participate.

Inclusion criteria were individuals aged 55 years or older who had resided in the Gasabo district for at least 6 months prior to the interview and were capable of providing informed consent. Trained nutritionists conducted the data collection, which included socio-demographic characteristics and dietary assessments. The socio-demographic variables recorded were age, sex, education level, employment status, marital status, family size and composition, religion, and socioeconomic status. The socioeconomic status was assessed by asking participants to report on the respective socioeconomic categories of their households. This classification exists in Rwanda where it is known as Ubudehe (38), and the categorization is based on a combination of different indicators, including household income, properties, and assets (38). This categorization has four levels designated as 1 = poorest, 2 = poor, 3 = rich, and 4 = richest (38).

### Estimation of food intake

Food intake data were collected through repeated 24-h recall interviews on randomly selected non-consecutive days. The selected days included both weekdays and weekends, with at least 15 days between recalls. All interviews were conducted in the local language (Kinyarwanda). Participants were asked to list all foods and beverages consumed the day before from waking up, until waking up on the day of the interview. Details about ingredients, preparation methods, mealtimes, brand names, quantities consumed, and any leftovers were recorded. Participants used household measurements such as plates, cups, and spoons to demonstrate portion sizes. Solid food portions were estimated by the interviewer using a kitchen scale (Clas Ohlson Kitchen Scale, Model: CFC2028), while liquid portions were measured with graduated cups. When participants had difficulty estimating portion sizes, a Kenyan food atlas (39) was also used for reference due to its similarity to Rwandan foods and the absence of a specific Rwandan tool.

### Estimation of nutrient intake

During the study, there was no available Food Composition Table (FCT) for Rwanda. Therefore, an FCT was compiled primarily based on the Kenyan Food Composition Table (40). For foods not covered by the Kenyan database, other reliable food databases were consulted (41–44). The average of the 2-day dietary recalls was used to compute nutrient intake. The nutrient calculations were conducted in the Compl-EAT software (version 1.0) from Wageningen University and Research, The Netherlands.

### The EAT-Lancet diet index (score)

The recorded food intake was assigned to the food groups in the EAT-Lancet diet (Supplemental tables 1 and 2). If there was uncertainty about which group to categorize the food in, it was discussed and consented among the authors (T.H. and A.S.). Based on the recorded food intake, we evaluated adherence to the EAT-Lancet diet using the score from Stubbendorff et al. (Supplemental Table 3) (23). This score has performed well in a systematic comparison to other EAT-Lancet scores in cohorts from Denmark, Sweden, and Mexico (22). Fourteen different food groups were assigned 0–3 points, resulting in a total score between 0 and 42 points. The tertiles (T1, T2, and T3) were created from the raw scores using the ‘xtile’ command in STATA. These raw scores and their corresponding tertile categories were then analyzed in relation to the intake of 13 micronutrients.

### Statistical analyses

The EAT-Lancet index was used as an indicator of the level of adherence to the EAT-Lancet recommendations. The EAT-Lancet index was modelled as a continuous variable and as tertiles. The baseline characteristics present the initial data of all participants and are categorized as tertiles based on their adherence to the EAT-Lancet diet. Continuous variables were summarized as means with SDs, and categorical variables were expressed as numbers (percentages). Following the guidelines for reporting descriptive statistics (45, 46) significance tests were omitted when describing the variability of participant characteristics.

Multivariable regression analyses were conducted, adjusting for the confounding variables of age and sex in model 1. In model 2, we further adjusted for total energy intake. Linear regression was applied for estimation of the marginal means for various nutrient intakes across the tertiles of adherence, and beta values and *P*-values were reported for the continuous variable (0–42 points) of the EAT-Lancet diet adherence scale.

The analyses were conducted using STATA Software (version 18.0, StataCorp, USA). A two-sided *P* < 0.05 was considered statistically significant.

### Ethical approvals

The study was conducted in accordance with Declaration of Helsinki principles for the research involving human subjects. The study received ethical approval from the Institutional Review Board (IRB) of the College of Medicine and Health Sciences, University of Rwanda (Ref. No: 291/CMHS IRB/2021), and the Regional Committee for Medical Research Ethics Western Norway (Ref. No: 163823). Additionally, permission to conduct the study was obtained from the authorities in Gasabo District (Ref. No: 1999/070102/2021). The study’s purpose was clearly explained to all participants, who then provided written informed consent.

## Results

### Characteristics of participants and adherence to the EAT-Lancet diet

A total of 334 older adults were included in the study (Supplemental Fig. 1). Participants’ ages ranged from 55 to 93 years, with a mean age of 67 years (Table 1). The majority lived with others, only 13% (*n* = 42) lived alone. Family sizes varied from 1 to 15 members, with a median size of four individuals. Over one-third (33%, *n* = 112) of the participants had no formal education, 42% (*n* = 139) had completed primary school, and 25% (*n* = 83) had secondary education or higher. The majority, 71% (*n* = 238) were unemployed. More than half of the participants (56%, *n* = 186) were in the poorest or poor wealth categories, while the remaining 44% (*n* = 148) were classified in the middle-income and only very few in the richest category.

**Table 1:** Baseline characteristics according to the tertiles of the EAT-Lancet diet score

	Tertiles of EAT-Lancet score			Total
	T1: 13–23	T2: 24–25	T3: 26–36	
*N*	100 (29.9%)	125 (37.4%)	109 (32.6%)	334 (100%)
Gender				
Male	31 (31.0%)	31 (24.8%)	18 (16.5%)	80 (24.0%)
Female	69 (69.0%)	94 (75.2%)	91 (83.5%)	254 (76.0%)
Age	66.4 (± 8.3)	66.9 (± 8.6)	66.4 (± 9.0)	66.6 (± 8.6)
Marital status				
Single/separated/divorced /widowed	41 (41.0%)	52 (41.6%)	37 (33.9%)	130 (38.9%)
Cohabiting/Married	59 (59.0%)	73 (58.4%)	72 (66.1%)	204 (61.1%)
Family conditions				
Live with other people in the HH	89 (89.0%)	106 (84.8%)	97 (89.0%)	292 (87.4%)
Alone in the household	11 (11.0%)	19 (15.2%)	12 (11.0%)	42 (12.6%)
Presence of other older adults in the HH				
Without any older adults	74 (74.0%)	86 (68.8%)	77 (70.6%)	237 (71.0%)
Living with other older adults	26 (26.0%)	39 (31.2%)	32 (29.4%)	97 (29.0%)
Education				
Never attended school	66 (66.0%)	85 (68.0%)	71 (65.1%)	222 (66.5%)
Attended school	34 (34.0%)	40 (32.0%)	38 (34.9%)	112 (33.5%)
Currently employed or working				
No	66 (66.0%)	87 (69.6%)	85 (78.0%)	238 (71.3%)
Yes	34 (34.0%)	38 (30.4%)	24 (22.0%)	96 (28.7%)
Religion				
Christian	93 (93.0%)	122 (97.6%)	109 (100%)	324 (97.0%)
Muslim	6 (6.0%)	1 (0.8%)	0 (0%)	7 (2.1%)
Others	1 (1.0%)	2 (1.6%)	0 (0%)	3 (0.9%)
Wealth category				
Category1/poorest	22 (22.0%)	36 (28.8%)	24 (22.0%)	82 (24.6%)
Category2/poor	31 (31.0%)	40 (32.0%)	33 (30.3%)	104 (31.1%)
Category3/middle income	45 (45.0%)	47 (37.6%)	52 (47.7%)	144 (43.1%)
Category4/rich	2 (2.0%)	2 (1.6%)	0 (0%)	4 (1.2%)

**Fig. 1:**
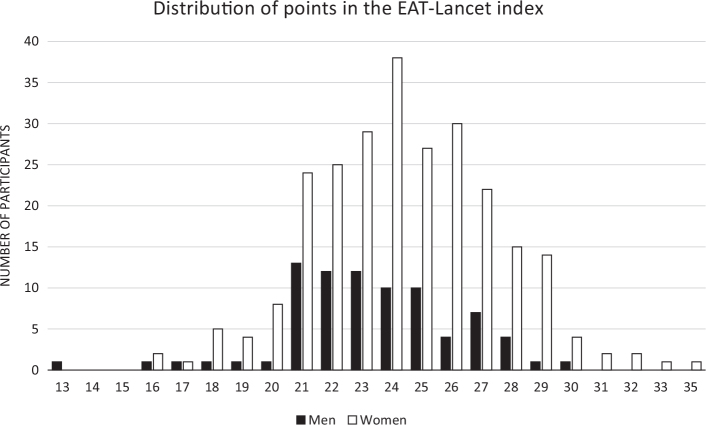
Distribution of the EAT-Lancet diet score among 80 men and 254 women in Kigali, Rwanda, based on data from repeated 24 h recalls. The maximum number of points was 42 indicating excellent adherence, and the score in this study population ranged from 13 to 36 with a median of 24.2. The score was categorized into three categories in the analyses (≤ 23, 24**–**25, ≥ 26).

When grouping the participants into tertiles (T1–T3) of adherence to the EAT-Lancet diet, there were no substantial differences in the baseline characteristics of the participants in the different tertiles, except that more women were in the highest tertile compared to men (Table 1). Also, there was a slight difference in religion, with religions other than Christianity being in the first or second tertile. The EAT-Lancet score ranged between 13 and 36, with a mean score of 24.2 (± 3.1). For women, the mean score was 24.4 (± 3.1) and for men slightly lower at 23.5 (± 3.1). The distribution of the score among men and women is outlined in Fig. 1.

### Adherence to the EAT-Lancet diet and nutritional characteristics of the diet

**Fig. 2:**
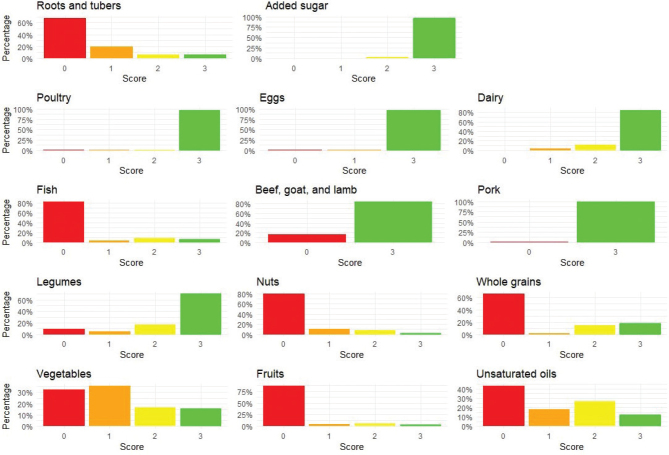
Proportion of participants (*n* = 334) with different scores across the 14 food groups in the EAT-Lancet index. A score of 0 indicates no adherence to the EAT-Lancet recommendation for that specific food group, while a score of 3 indicates full adherence (total possible score ranges from 0 to 42 points).

Adherence to the EAT-Lancet diet was associated with a noticeable increase in the consumption of whole grains and vegetables (Table 2). Conversely, the intake of roots and tubers, dairy, and red meat (including beef, lamb, and goat) decreased as adherence increased. The intake of fish and nuts also rose with higher adherence, indicating a shift towards these plant-based and aquatic protein sources in more adherent individuals. In contrast, the consumption of poultry and pork decreased, with nearly zero intake in the highest tertile. Legumes showed relatively stable intake across all tertiles, suggesting that they remain a consistent part of the diet regardless of adherence level. Additionally, the intake of unsaturated oils increased as adherence to the EAT-Lancet diet rose while the intake of added sugars remained relatively stable across all tertiles. Similarly, the participants had a low score in the EAT-Lancet index for the food groups roots/tubers, fish, nuts, whole grains, vegetables, fruit, and unsaturated oils (Fig. 2). They adhered most to the groups administered sugar, poultry, eggs, dairy, beef/goat/lamb, pork, and legumes.

**Table 2:** Daily food intake according to the adherence to the EAT-Lancet diet in mean (SD) g/day

	Tertiles of EAT-Lancet score			All
	T1: 13–23	T2: 24–25	T3: 26–36	
Wholegrains	44 (118)	103 (166)	134 (172)	95 (159)
Roots and tubers	437 (294)	320 (211)	275 (233)	340 (254)
Vegetables	97 (93)	147 (104)	255 (133)	167 (128)
Fruit	13 (52)	25 (61)	34 (79)	24 (65)
Dairy	126 (208)	78 (138)	72 (130)	90 (161)
Beef, lamb, goat	17 (42)	8.3 (26)	4 (16)	9.5 (29)
Pork	5.1 (32)	0 (0)	0 (0)	1.5 (18)
Poultry	4.1 (18)	2.7 (16)	0 (0)	2.2 (14)
Eggs	1.5 (11)	2 (11)	0.2 (2.4)	1.3 (9)
Fish	1.1 (8)	4.4 (13)	15 (32)	6.8 (21)
Legumes	113 (70)	134 (82)	137 (77)	128 (78)
Nuts	1.4 (5.6)	4.1 (8.9)	14 (28)	6.5 (18)
Unsaturated oils	7.2 (10)	18 (18)	27 (18)	18 (18)
Added sugar	7.7 (18)	7.1 (9.2)	6.9 (9.5)	7.2 (13)

Nutritional characteristics according to the EAT-Lancet diet scores are presented in Table 3. Higher EAT-Lancet scores were positively associated with energy intake. Dietary intake, measured in grams per day, showed a positive association between the EAT-Lancet score and protein (31 g/d in T1 vs. 36 g/d in T3), and fat (27 g/d in T1 vs. 50 g/d in T3). In contrast, carbohydrate intake exhibited a negative association although it was not significant (181 g/d in T1 vs. 169 g/d in T3). Measured as energy percentage (E%), there was a positive association with fat (9 E% in T1 vs. 15 E% in T3 vs.), a negative association with carbohydrates (64 E% in T1 vs. 51 E% in T3), while no significant association was seen for protein. As adherence to the EAT-Lancet diet increased, there was a notable rise in the intake in the majority of the studied micronutrients, and the association was stronger even after adjustment for energy intake (Table 3). Retinol-equivalents intake was more than doubled from the lowest tertile to the highest tertile (480 μg/d; 1,041 μg/d), with a significant association (β = 77.7, *P* < 0.001). Positive trends with higher intakes were also seen for thiamine, niacin, folate, vitamin B12, and vitamin C. No association with higher intake at higher adherence was seen for riboflavin. For minerals and trace elements, higher adherence to the EAT-Lancet diet was associated with a higher intake of calcium, phosphorus, magnesium, potassium, iron, and zinc. When further adjusted for energy intake (Table 3), we found that higher adherence to the EAT-Lancet diet was associated with a higher intake of retinol-equivalents, thiamine, folate, vitamin B12, vitamin C, calcium, phosphorous, magnesium, and iron. However, there was a negative association with riboflavin, though the difference was minor in magnitude. There were no significant associations for niacin, potassium, and zinc.

**Table 3:** Nutrient intake across tertiles of EAT-Lancet score

	EAT-Lancet diet score				
	Tertiles			Continuous, 0–42 points	
	T1: 13–23	T2: 24–25	T3: 26–36	β	*P*
Energy (kcal)	1,137 (855)	1,193 (762)	1,342 (820)	32.52	< 0.001
Carbohydrates g/day	180.6 (130.6)	167.5 (116.3)	168.6 (125.2)	0.12	0.924
Carbohydrates E%	63.8% (23.1%)	57.7% (20.6%)	50.8% (22.1%)	-0.01	< 0.001
Fat g/day	26.7 (46.6)	36.9 (41.5)	50.4 (44.7)	3.00	< 0.001
Fat E%	9.2% (9.9%)	11.8% (8.8%)	14.8% (9.5%)	0.01	< 0.001
Protein g/day	30.8 (29.9)	30.3 (26.6)	35.5 (28.6)	0.46	0.112
Protein E%	10.7% (6.1%)	10.2% (5.4%)	10.8% (5.8%)	0.00	0.238
Animal-sourced protein g/day	11 (24.2)	6.4 (21.5)	7.1 (23.2)	-0.77	0.001
Animal-sourced protein E%	3.4% (6.4%)	1.9% (5.7%)	2.1% (6.1%)	0.00	< 0.001
Plant based protein g/day	19.5 (16.4)	23.6 (14.6)	28.3 (15.7)	1.24	< 0.001
Plant based protein E%	7.2% (4.6%)	8.2% (4.1%)	8.7% (4.4%)	0.00	< 0.001
**Model 1 (non-energy adj)**					
Retinol-equivalents (μg)	480 (1,310)	666 (1,167)	1,041 (1,256)	77.7	< 0.001
Thiamine (mg)	0.44 (0.44)	0.52 (0.39)	0.59 (0.42)	0.02	< 0.001
Riboflavin (mg)	0.71 (0.94)	0.66 (0.84)	0.77 (0.9)	0.00	0.955
Niacin (mg)	6.52 (7.16)	6.69 (6.38)	8.59 (6.87)	0.26	< 0.001
Folate (μg)	201 (174)	231 (155)	272 (167)	9.85	< 0.001
Vitamin B12 (μg)	1.68 (8.22)	1.76 (7.33)	3.98 (7.88)	0.32	< 0.001
Vitamin C (mg)	66.2 (100)	72 (89.4)	97.3 (96.2)	4.97	< 0.001
Calcium (mg)	533 (718)	573 (640)	787 (689)	36.17	< 0.001
Phosphorus (mg)	757 (709)	796 (632)	994 (680)	32.0	< 0.001
Magnesium (mg)	236 (225)	283 (201)	345 (216)	15.8	< 0.001
Potassium (mg)	2,435 (1,782)	2,324 (1,588)	2,589 (1,709)	36.6	0.033
Iron (mg)	11.1 (11.3)	13.2 (10.1)	16.4 (10.9)	0.73	< 0.001
Zinc (mg)	5.4 (5.4)	5.3 (4.8)	6.6 (5.2)	0.14	0.010
**Model 2 (energy adj)**					
Retinol-equivalents (μg)	533 (1,211)	686 (1,075)	970 (1,167)	58.56	< 0.001
Thiamine (mg)	0.47 (0.32)	0.53 (0.28)	0.55 (0.31)	0.01	0.012
Riboflavin (mg)	0.76 (0.82)	0.68 (0.72)	0.70 (0.79)	-0.02	0.020
Niacin (mg)	7.03 (5.19)	6.88 (4.6)	7.91 (5)	0.07	0.203
Folate (μg)	212 (135)	236 (120)	256 (130)	5.67	< 0.001
Vitamin B12 (μg)	1.85 (8.1)	1.83 (7.19)	3.75 (7.81)	0.25	< 0.001
Vitamin C (mg)	70 (94)	73.4 (83.4)	92.2 (90.6)	3.62	< 0.001
Calcium (mg)	566 (648)	584 (575)	743 (625)	24.23	< 0.001
Phosphorus (mg)	803 (559)	812 (496)	934 (539)	15.23	0.006
Magnesium (mg)	250 (184)	288 (163.2)	326 (177)	10.95	< 0.001
Potassium (mg)	2,556 (1,342)	2,369 (1,191)	2,427 (1,293)	-8.44	0.522
Iron (mg)	11.8 (9.7)	13.4 (8.6)	15.5 (9.3)	0.51	< 0.001
Zinc (mg)	5.7 (4.2)	5.4 (3.7)	6.2 (4)	0.00	0.969

Model is based on linear regression adjusted for age and sex, with estimated marginal means. The coefficient (β) represents the increase in nutrient intake for each 1-point increase in the EAT-Lancet diet score.

## Discussion

This study assessed the adherence to the EAT-Lancet diet and its impact on micronutrients among older adults in Kigali, Rwanda. The study found that higher adherence to the EAT-Lancet diet was associated with higher intakes of most micronutrients; retinol-equivalents, thiamine, folate, niacin, vitamin B12, vitamin C, calcium, phosphorus, magnesium, potassium, iron, and zinc. These differences persisted even after adjustment for energy intake. Higher adherence to the EAT-Lancet diet might therefore reflect a more nutrient-rich diet in this population.

Our study is the first to study the relationship between the adherence to the EAT-Lancet diet and micronutrient intake in the older population in a sub-Saharan setting. We observed that even participants in the highest tertile of adherence to the EAT-Lancet diet still did not meet the recommendations of the EAT-Lancet reference diet, as expressed by the overall mean score of 24.4 for women and 23.5 for men, with 42 points expressing full adherence. The EAT-Lancet diet, designed for global targets, has been criticized as being low in micronutrients. Thus, the nutritional adequacy of this diet, which emphasizes plant-based foods and significantly restricts animal products, remains a subject of debate. There is ongoing discussion about whether this diet can adequately meet the nutritional needs of diverse populations (47–49), and only a limited number of studies have explored this issue.

The findings of this study highlight specific dietary sources of key micronutrients within the context of EAT-Lancet diet adherence. Low intake of riboflavin might be related to the limited dairy intake in this population. Furthermore, reported thiamine and niacin intakes were far below the dietary reference intake levels, even though both increased with greater adherence to the EAT-Lancet diet. This underscores the need for expanded research on micronutrient status beyond vitamin A and iron in sub-Saharan African populations, particularly for nutrients less frequently studied, such as riboflavin, thiamine, and niacin. Interestingly, while riboflavin initially showed no significant association with adherence to the EAT-Lancet dietary pattern, a modest negative association appeared after adjusting for energy intake. This shift primarily highlights differences in nutrient density, as higher adherence tends toward a plant-focused dietary pattern, naturally lower in certain animal-based riboflavin sources like dairy. These findings suggest a valuable opportunity for optimizing the EAT-Lancet dietary recommendations by intentionally incorporating riboflavin-rich plant-based foods or fortified alternatives. Doing so can enhance nutritional adequacy while maintaining the recognized health and environmental benefits of the EAT-Lancet diet, particularly in regions like Rwanda, where traditional diets heavily depend on plant-based foods for specific nutrients. Similarly, niacin, potassium, and zinc, which initially had positive associations, became non-significant with energy adjustment. This shift suggests that the associations observed for these nutrients were largely influenced by overall energy intake rather than dietary adherence itself. Adjusting for energy intake may therefore highlight the fact that higher adherence to the EAT-Lancet diet does not necessarily guarantee increased intake of all individual micronutrients, pointing to a need for careful consideration of specific nutrient sources within the diet.

Indeed, a Swedish study has shown that adjusting for energy intake in dietary GHG emissions assessments significantly alters associations with micronutrient intake, often reversing or weakening observed relationships (50). The EAT-Lancet diet is based on specific recommendations for absolute food intake amounts, and the implications of energy adjustments in studies on this topic need to be explored to a greater extent. The EAT-Lancet diet score used in this study assigns higher points for increased intake in seven food groups and deducts points for higher intake in another seven. As a result, higher adherence does not necessarily correspond to an increase or decrease in overall food intake.

To our knowledge, only two studies have studied micronutrient intake in relation to the EAT-Lancet diet, based on observational data. A study by Kesse-Guyot et al. examined the environmental and nutritional impacts of adhering to the EAT-Lancet diet among French adults, using the EAT-Lancet Diet Index (ELD-I) as a scoring system (51). The findings highlighted significant improvements in the intake of several key micronutrients among those with higher adherence to the diet. Participants with higher adherence to the EAT-Lancet diet demonstrated increased intake of vitamins such as vitamin C, folate, and vitamin E. The study also noted that while participants with higher adherence had better overall nutrient profiles, they consumed lower quantities of heme-iron, which is more readily absorbed by the body compared to non-heme iron found in plant sources. However, the study pointed out that despite the lower intake of heme-iron, the overall iron intake among high adherents was still adequate due to the inclusion of plant-based sources of iron. Overall, the study concluded that adherence to the EAT-Lancet diet generally led to an improved intake of several important vitamins and minerals, especially those associated with plant-based foods. The second study, conducted in Germany, used yet another method for quantifying the adherence to the EAT-Lancet diet (52). In this study, higher adherence was associated with lower intake of protein, added sugar, and cholesterol, but higher fiber intake. There was a significant increase in magnesium, vitamin E, folic acid, and vitamin K with higher adherence.

Despite the clear nutritional and environmental advantages associated with the EAT-Lancet dietary pattern, concerns remain about potential conflicts between sustainability objectives and micronutrient sufficiency, particularly among vulnerable groups such as older populations in low-income settings. Animal-derived foods, reduced in EAT-Lancet diets, are traditionally key sources of bioavailable micronutrients like riboflavin, zinc, and heme-iron, which are especially crucial for older adults due to age-related changes in absorption and metabolism. Reducing these foods may thus exacerbate existing micronutrient deficiencies unless careful planning and targeted interventions are implemented. However, it is important to recognize that this dietary shift also offers considerable opportunities for nutritional synergy. Plant-based diets, aligned with EAT-Lancet recommendations, are typically rich in vitamins, minerals, dietary fiber, and bioactive compounds beneficial for chronic disease prevention and management. Moreover, promoting diverse, nutrient-dense plant foods and fortified alternatives can effectively mitigate potential deficiencies. This approach can align nutritional adequacy with environmental sustainability goals, creating positive health outcomes alongside reduced environmental impacts. Thus, achieving optimal outcomes requires integrated strategies, including education, targeted dietary guidance, and enhanced accessibility to nutrient-rich, sustainable food sources tailored to local contexts.

### Strengths and limitations

This study has several strengths. Firstly, dietary intake was measured by two non-consecutive dietary interviews in the local language. This method also does not require the participants to be literate, and potential misinterpretations are minimized, as participants can directly clarify their responses with the interviewer, reducing errors and missing data. The 24-h recall offers advantages, such as capturing a broader range of foods that might be missed by a more restrictive questionnaire. Secondly, the study applied a sampling scheme that makes the selected population representative. Thirdly, our study used a validated EAT Lancet score that has been shown to be robust in other LMICs also. The EAT-Lancet scoring system is widely regarded as an improvement over other tools like dietary diversity score that have been used to predict the adequacy of micronutrient intake in LMICs by counting food groups (53). Unlike its predecessors, EAT-Lancet dietary pattern is 1) designed to meet the nutritional requirements of both adults and young children older than 2 years, and 2) uses the quantity of food consumed to predict nutrient adequacy while also considering environmental sustainability (23). Lastly, the associations between almost all micronutrients assessed and the EAT-Lancet diet score were robust when further adjusting for energy intake of the participants (22). However, the study also some limitations. Although macronutrient estimates obtained from 24-h recalls are generally more reliable, estimates for vitamins and minerals tend to be less consistent (54). This questionnaire also focused on dietary intake and no data were collected on micronutrient supplementation. However, using the micro-nutrient supplement is not common among adults in Rwanda, and at the time of data collection, there was no known micronutrient supplementation program among the older population in Rwanda. We cannot rule out misreporting or residual confounding. Data were obtained in one season and thus may not reflect seasonal variations in food intake. Moreover, the data presented in this study were also collected during the COVID-19 pandemic with intermittent lockdowns, and these factors might have also contributed to low quantity and diversity of consumed foods. Additionally, the use of a Kenyan food database might not accurately reflect the nutritional intake, as a Rwandan food table. Analytically, the model did not include formal diagnostic tests for all assumptions of linear regression as the study primarily focused on the associations rather than predictions. Lastly, given the generally low adherence to the EAT-Lancet diet in this cohort, our analyses focus on the linear associations between the adherence score and micronutrient intake, which reflect the relative ranking of participants rather than strict adherence thresholds. This approach accounts for potential underreporting and highlights how incremental increases in adherence may influence nutrient intake.

### Future studies

There is a need for studies that assess not only the micronutrient intake of the population but also micronutrient status. It has been emphasized that both nutrient intake and status should be considered when evaluating food sustainability, especially given the lower bioavailability of micronutrients in plant-based foods (55). As an example, the EAT-Lancet diet, which is predominantly plant-based, may result in non-heme iron being the primary source of iron, a form with lower bioavailability. This is particularly important given the already high prevalence of anemia in the Rwandan population (56). Further, there should be more focus on understudied micronutrients, such as thiamine, riboflavin, and niacin, which had a very low intake in the current population.

## Conclusion

The findings indicate that higher adherence to the EAT-Lancet diet, which emphasizes plant-based foods and limits animal products, is associated with a higher intake of key micronutrients such as retinol-equivalents, thiamine, folate, vitamin B12, calcium, and vitamin C among older adults in Kigali, Rwanda. The findings of this study also underscore the need for considering local dietary patterns and food availability when implementing global dietary guidelines like the EAT-Lancet diet.

## Supplementary Material



## Supporting Materials

**Supplemental table 1:** The proposed EAT-Lancet diet by Willett et al., ‘Food in the Anthropocene: the EAT-Lancet Commission on healthy diets from sustainable food systems’

**Supplemental table 2:** Description of included food components for the components in the EAT-Lancet diet index.

**Supplemental table 3:** Criteria for the EAT-Lancet index developed by Stubbendorff et al (23). All amounts are dry (uncooked) weight.

**Supplementary Figure 1:** Flowchart for the selection of the participants

## References

[1] Xu X, Sharma P, Shu S, Lin T-S, Ciais P, Tubiello FN, et al. Global greenhouse gas emissions from animal-based foods are twice those of plant-based foods. Nat Food 2021; 2(9): 724–32. doi: 10.1038/s43016-021-00358-x 37117472

[2] Gerber PJ, Steinfeld H, Henderson B, Mottet A, Opio C, Dijkman J, et al. Tackling climate change through livestock: a global assessment of emissions and mitigation opportunities. Rome: Food and Agriculture Organization of the United Nations (FAO); 2013. Available from: https://www.fao.org/4/i3437e/i3437e.pdf [cited 17 September 2024].

[3] Sutton WR, Lotsch A, Prasann A. Recipe for a livable planet: achieving net zero emissions in the agrifood system. Washington, DC: World Bank Publications; 2024.

[4] Clonan A, Roberts KE, Holdsworth M. Socioeconomic and demographic drivers of red and processed meat consumption: implications for health and environmental sustainability. Proc Nutr Soc 2016; 75(3): 367–73. doi: 10.1017/S0029665116000100 27021468 PMC4974628

[5] Popkin BM. The nutrition transition in low-income countries: an emerging crisis. Nutr Rev 1994; 52(9): 285–98. doi: 10.1111/j.1753-4887.1994.tb01460.x 7984344

[6] Latino LR, Pica-Ciamarra U, Wisser D. Africa: the livestock revolution urbanizes. Glob Food Sec 2020; 26: 100399. doi: 10.1016/j.gfs.2020.100399 33052301 PMC7543768

[7] Bongaarts J. Development: slow down population growth. Nature 2016; 530(7591): 409–12. doi: 10.1038/530409a 26911766

[8] Kearney J. Food consumption trends and drivers. Philos Trans R Soc B Biol Sci 2010; 365(1554): 2793–807. doi: 10.1098/rstb.2010.0149 PMC2935122 20713385

[9] World Health Organization. Decade of healthy ageing: baseline report. Geneva: WHO; 2020. Available from: https://www.who.int/publications/i/item/9789240017900 [cited 23 June 2024].

[10] Beal T, Massiot E, Arsenault JE, Smith MR, Hijmans RJ. Global trends in dietary micronutrient supplies and estimated prevalence of inadequate intakes. PLoS One 2017; 12(4): e0175554. doi: 10.1371/journal.pone.0175554 28399168 PMC5388500

[11] Passarelli S, Free CM, Shepon A, Beal T, Batis C, Golden CD. Global estimation of dietary micronutrient inadequacies: a modelling analysis. Lancet Glob Health 2024; 12(10): e1590–9. doi: 10.1016/S2214-109X(24)00276-6 39218000 PMC11426101

[12] Stevens GA, Beal T, Mbuya MNN, Luo H, Neufeld LM, Addo OY, et al. Micronutrient deficiencies among preschool-aged children and women of reproductive age worldwide: a pooled analysis of individual-level data from population-representative surveys. Lancet Glob Health 2022; 10(11): e1590–9. doi: 10.1016/S2214-109X(22)00367-9 36240826 PMC10918648

[13] Obeng P, Kyereh HK, Sarfo JO, Ansah EW, Attafuah PYA. Nutritional status and associated factors of older persons in sub-Saharan Africa: a scoping review. BMC Geriatr 2022; 22(1): 416. doi: 10.1186/s12877-022-03062-y 35545755 PMC9097054

[14] Crippa M, Solazzo E, Guizzardi D, Monforti-Ferrario F, Tubiello FN, Leip A. Food systems are responsible for a third of global anthropogenic GHG emissions. Nat Food 2021; 2(3): 198–209. doi: 10.1038/s43016-021-00225-9 37117443

[15] Shukla PR, Skea J, Slade R, Al Khourdajie A, van Diemen R, McCollum D, et al. Climate change 2022: mitigation of climate change. Contribution of Working Group III to the Sixth Assessment Report of the Intergovernmental Panel on Climate Change. Cambridge: Cambridge University Press; 2022.

[16] Willett W, Rockström J, Loken B, Springmann M, Lang T, Vermeulen S, et al. Food in the Anthropocene: the EAT-Lancet Commission on healthy diets from sustainable food systems. Lancet 2019; 393(10170): 447–92. doi: 10.1016/S0140-6736(18)31788-4 30660336

[17] Benton TG, Bieg C, Harwatt H, Pudasaini R, Wellesley L. Food system impacts on biodiversity loss. Three levers for food system transformation in support of nature. London: Chatham House; 2021.

[18] Scarborough P, Clark M, Cobiac LJ, Papier K, Knuppel A, Lynch J, et al. Vegans, vegetarians, fish‑eaters and meat‑eaters in the UK show discrepant environmental impacts. Nat Food 2023; 4(7): 565–74. doi: 10.1038/s43016-023-00795-w 37474804 PMC10365988

[19] Food and Agriculture Organization of the United Nations (FAO). Our actions are our future: healthy diets for a #ZeroHunger world. 1st ed. Rome: FAO; 2019, 12 p. Available from: https://openknowledge.fao.org/handle/20.500.14283/ca5268en [cited August 2024].

[20] Schneider KR, Fanzo J, Haddad L, Herrero M, Moncayo JR, Herforth A, et al. The state of food systems worldwide in the countdown to 2030. Nat Food 2023; 4(12): 1090–110. doi: 10.1038/s43016-023-00885-9 38114693 PMC10730405

[21] Bui LP, Pham TT, Wang F, Chai B, Sun Q, Hu FB, et al. Planetary health diet index and risk of total and cause-specific mortality in three prospective cohorts. Am J Clin Nutr 2024; 120(1): 80–91. doi: 10.1016/j.ajcnut.2024.03.019 38960579 PMC11251201

[22] Stubbendorff A, Stern D, Ericson U, Sonestedt E, Hallström E, Borné Y, et al. A systematic evaluation of seven different scores representing the EAT-Lancet reference diet and mortality, stroke, and greenhouse gas emissions in three cohorts. Lancet Planet Health 2024; 8(6): e391–401. doi: 10.1016/S2542-5196(24)00094-9 38849181

[23] Stubbendorff A, Sonestedt E, Ramne S, Drake I, Hallström E, Ericson U. Development of an EAT-Lancet index and its relation to mortality in a Swedish population. Am J Clin Nutr 2022; 115(3): 705–16. doi: 10.1093/ajcn/nqab369 34791011 PMC8895215

[24] Zhang S, Stubbendorff A, Olsson K, Ericson U, Niu K, Qi L, et al. Adherence to the EAT-Lancet diet, genetic susceptibility, and risk of type 2 diabetes in Swedish adults. Metabolism 2023; 141: 155401. doi: 10.1016/j.metabol.2023.155401 36682448

[25] Zhang S, Dukuzimana J, Stubbendorff A, Ericson U, Borné Y, Sonestedt E. Adherence to the EAT-Lancet diet and risk of coronary events in the Malmo Diet and Cancer cohort study. Am J Clin Nutr 2023; 117(5): 903–9. doi: 10.1016/j.ajcnut.2023.02.018 36841443

[26] Stanton AV. Plant-based diets–impacts of consumption of little or no animal-source foods on human health. Front Nutr 2024; 11: 1423925. doi: 10.3389/fnut.2024.1423925 39360272 PMC11444979

[27] Nicol K, Nugent AP, Woodside JV, Hart KH, Bath SC. Iodine and plant-based diets: a narrative review and calculation of iodine content. Br J Nutr 2024; 131(2): 265–75. doi: 10.1017/S0007114523001873 37622183 PMC10751939

[28] Alvarez-Nuncio MDC, Ziegler TR. Micronutrient status and protein-energy malnutrition in free-living older adults: a current perspective. Curr Opin Gastroenterol 2024; 40(2): 99–105. doi: 10.1097/MOG.0000000000001000 38193299 PMC10872245

[29] Ali Z, Scheelbeek PFD, Felix J, Jallow B, Palazzo A, Segnon AC, et al. Adherence to EAT-Lancet dietary recommendations for health and sustainability in the Gambia. Environ Res Lett 2022; 17(10): 104043. doi: 10.1088/1748-9326/ac9326 36238079 PMC9536464

[30] Trijsburg L, Talsma EF, Crispim SP, Garrett J, Kennedy G, de Vries JHM, et al. Method for the development of WISH, a globally applicable index for healthy diets from sustainable food systems. Nutrients 2020; 13(1): 93. doi: 10.3390/nu13010093 33396659 PMC7824146

[31] Keding GB, Sarfo J, Pawelzik E. Healthy diets from sustainable food systems: calculating the WISH scores for women in rural East Africa. Nutrients 2023; 15(12): 2699. doi: 10.3390/nu15122699 37375603 PMC10302709

[32] Sharma M, Kishore A, Roy D, Joshi K. A comparison of the Indian diet with the EAT-Lancet reference diet. BMC Public Health 2020; 20(1): 812. doi: 10.1186/s12889-020-08951-8 32471408 PMC7260780

[33] National Institute of Statistics of Rwanda (NISR). Key figures: 5th Rwanda population and housing census (PHC). Kigali: NISR; 2022. Available from: https://www.statistics.gov.rw/publication/key-figures-5th-rwanda-population-and-housing-census-phc [cited February 2023].

[34] Food and Agriculture Organization of the United Nations (FAO). Food balances. FAOSTAT; 2020. Available from: https://www.fao.org/faostat/en/#data/FBS/report [cited March 2023].

[35] Habumugisha T, Matsiko E, Måren IE, Kaiser M, Melse-Boonstra A, Borgonjen-van den Berg K, et al. Protein intake and muscle mass of community-dwelling older adults: a cross-sectional study in Kigali, Rwanda. Sci Rep 2025; 15(1): 1–13. doi: 10.1038/s41598-025-03291-2 40413214 PMC12103568

[36] Habumugisha T, Måren IE, Matsiko E, Kaiser M, Dierkes J, Engebretsen IM. Older adults’ perceptions about meat consumption: a qualitative study in Gasabo district, Kigali, Rwanda. BMC Public Health 2024; 24(1): 1515. doi: 10.1186/s12889-024-19038-z 38840236 PMC11155052

[37] Habumugisha T, Engebretsen IMS, Måren IE, Kaiser CWM, Dierkes J. Reducing meat and/or dairy consumption in adults: a systematic review and meta-analysis of effects on protein intake, anthropometric values, and body composition. Nutr Rev 2024; 82(3): 277–301. doi: 10.1093/nutrit/nuad055 37236631 PMC10859689

[38] Ezeanya C. Home-grown and grassroots-based strategies for determining inequality towards policy action: Rwanda’s Ubudehe approach in perspective. WIDER Working Paper. Helsinki: The United Nations University World Institute for Development Economics Research (UNU-WIDER); 2015. doi: 10.35188/UNU-WIDER/2015/893-3

[39] Walsh H, Anono E, Okoth V, Clinton B, Mubasu D, Hyvönen A, et al. Photographic food atlas. 2018. Available from: https://kenfinedura.com/wp-content/uploads/2020/03/1st-version-photographic-food-atlas-30.07.2018-1.pdf [Cited May 2024].

[40] FAO/Government of Kenya. Kenya food composition tables. Nairobi: FAO/Government of Kenya; 2018, p. 254.

[41] Vincent A, Grande F, Compaoré E, Amponsah Annor G, Addy PA, Aburime LC, et al. FAO/INFOODS Food Composition Table for Western Africa (2019) user guide & condensed food composition table / Table de composition des aliments FAO/INFOODS pour l’Afrique de l’Ouest (2019) Guide d’utilisation & table de composition des aliments condensée. Rome: Food and Agriculture Organization of the United Nations (FAO); 2020. Available from: https://openknowledge.fao.org/server/api/core/bitstreams/51ca3ee4-9bd4-4077-b746-93cecd34b2c0/content [cited 6 August 2025].

[42] Lukmanji Z, Hertzmark E, Mlingi N, Assey V, Ndossi G, Fawzi W. Tanzania food composition tables. Dar Es Salaam: MUHAS-TFNC, HSPH; 2008.

[43] Wolmarans P, Danster N, Dalton A, Rossouw K, Schönfeldt H. Condensed food composition tables for South Africa. Cape Town: Medical Research Council; 2010, p. 1–126.

[44] U.S. Department of Agriculture, Agricultural Research Service. National nutrient database for standard reference, release 28. 2015. Available from: http://www.ars.usda.gov/ba/bhnrc/ndl [cited November 2023].

[45] Hayes-Larson E, Kezios KL, Mooney SJ, Lovasi G. Who is in this study, anyway? Guidelines for a useful Table 1. J Clin Epidemiol 2019; 114: 125–32. doi: 10.1016/j.jclinepi.2019.06.011 31229583 PMC6773463

[46] Vandenbroucke JP, von Elm E, Altman DG, Gotzsche PC, Mulrow CD, Pocock SJ, et al. Strengthening the Reporting of Observational Studies in Epidemiology (STROBE): explanation and elaboration. Int J Surg 2014; 12(12): 1500–24. doi: 10.1016/j.ijsu.2014.07.014 25046751

[47] Tucci M, Martini D, Del Bo’ C, Marino M, Battezzati A, Bertoli S, et al. An Italian-Mediterranean dietary pattern developed based on the EAT-Lancet Reference Diet (EAT-IT): a nutritional evaluation. Foods 2021; 10(3): 558. doi: 10.3390/foods10030558 33800396 PMC8002105

[48] Berthy F, Brunin J, Allès B, Reuzé A, Touvier M, Hercberg S, et al. Higher adherence to the EAT-Lancet reference diet is associated with higher nutrient adequacy in the NutriNet-Santé cohort: a cross-sectional study. Am J Clin Nutr 2023; 117(6): 1174–85. doi: 10.1016/j.ajcnut.2023.03.029 37019361

[49] Beal T, Ortenzi F, Fanzo J. Estimated micronutrient shortfalls of the EAT–Lancet planetary health diet. Lancet Planetary Health 2023; 7(3): e233–7. doi: 10.1016/S2542-5196(23)00006-2 36889864

[50] Stubbendorff A, Hallström E, Tomova G, Borné Y, Janzi S, Sonestedt E, et al. Greenhouse gas emissions in relation to micronutrient intake and implications of energy intake: a comparative analysis of different modeling approaches. Am J Clin Nutr 2025; 121(5): 1063–76. doi: 10.1016/j.ajcnut.2025.02.031 40074038 PMC12107493

[51] Kesse-Guyot E, Rebouillat P, Brunin J, Langevin B, Allès B, Touvier M, et al. Environmental and nutritional analysis of the EAT-Lancet diet at the individual level: insights from the NutriNet-Santé study. J Clean Prod 2021; 296: 126555. doi: 10.1016/j.jclepro.2021.126555

[52] Montejano Vallejo R, Schulz CA, van de Locht K, Oluwagbemigun K, Alexy U, Nöthlings U. Associations of adherence to a dietary index based on the EAT-lancet reference diet with nutritional, anthropometric, and ecological sustainability parameters: results from the German DONALD Cohort Study. J Nutr 2022; 152(7): 1763–72. doi: 10.1093/jn/nxac094 35554563 PMC9258554

[53] Nithya DJ, Bhavani RV. Factors which may limit the value of dietary diversity and its association with nutritional outcomes in preschool children in high burden districts of India. Asia Pac J Clin Nutr 2018; 27(2): 413–20. 29384331 10.6133/apjcn.032017.23

[54] Gibson RS, Charrondiere UR, Bell W. Measurement errors in dietary assessment using self-reported 24-hour recalls in low-income countries and strategies for their prevention. Adv Nutr 2017; 8(6): 980–91. doi: 10.3945/an.117.016980 29141979 PMC5683000

[55] McLaren S, Berardy A, Henderson A, Holden N, Huppertz T, Jolliet O, et al. Integration of environment and nutrition in life cycle assessment of food items: opportunities and challenges. Rome: Food and Agriculture Organization of the United Nations (FAO); 2021, 161 p. doi: 10.4060/cb8054en

[56] Global Nutrition Report. Rwanda country nutrition profile. 2023. Available from: https://globalnutritionreport.org/resources/nutrition-profiles/africa/eastern-africa/rwanda/ [cited 7 November 2024].

