# Supplementary material for: Adherence to the EAT-lancet dietary pattern among older adults in Rwanda and its association with micronutrient intake
Source: Food Nutr Res. 2025 Aug 18;69:10.29219/fnr.v69.12174. doi: 10.29219/fnr.v69.12174 (PMC12413755; doi:10.29219/fnr.v69.12174)
Supplement: Supplementary file 1 [file FNR-69-12174-s1.docx]

Supplemental table 1: The proposed EAT-Lancet diet by Willett et al., “Food in the Anthropocene: the EAT-Lancet Commission on healthy diets from sustainable food systems” [1].

|  |  | Macronutrient intake (possible range), g/day | Caloric intake, kcal/day | |  | |
| --- | --- | --- | --- | --- | --- | --- |
|  | **Whole grains** |  |  | |  | |
|  | Rice, wheat, corn and other† | 232 (total grains 0-60% of energy) | 811 | |  | |
|  | **Tubers or starchy vegetables** |  |  | |  | |
|  | Potatoes and casava | 50 (0-100) | 39 | |  | |
|  | **Vegetables** |  |  | |  | |
|  | All vegetables | 300 (200-600) |  | |  | |
|  | Dark green vegetables | 100 | 23 | |  | |
|  | Red and orange vegetables | 100 | 30 | |  | |
|  | Other vegetables | 100 | 25 | |  | |
|  | **Fruits** |  |  | |  | |
|  | All fruit | 200 (100-300) | 126 | |  | |
|  | **Dairy foods** |  |  | |  | |
|  | Whole milk or derivative equivalents (eg, cheese) | 250 (0-500) | 153 | |  | |
|  | **Protein sources**‡ |  |  | |  | |
|  | Beef and lamb | 7 (0-14) | 15 | |  | |
|  | Pork | 7 (0-14) | 15 | |  | |
|  | Chicken and other poultry | 29 (0-58) | 62 | |  | |
|  | Eggs | 13 (0-25) | 19 | |  | |
|  | Fish | 28 (0-100) | 40 | |  | |
|  | Legumes |  |  | |  | |
|  | Dry beans, lentils and peas | 50 (0-100) | 172 | |  | |
|  | Soy foods | 25 (0-50) | 112 | |  | |
|  | Peanuts | 25 (0-75) | 142 | |  | |
|  | Tree nuts | 25 | 149 | |  | |
|  | **Added fats** |  |  | |  | |
|  | Palm oil | 6.8 (0-6.8) | 60 | |  | |
|  | Unsaturated oils¶ | 40 (20-80) | 354 | |  | |
|  | Dairy fats (included in milk) | 0 | 0 | |  | |
|  | Lard or tallow║ | 5 (0-5) | 36 | |  | |
|  | **Added sugars** |  |  | |  | |
|  | All sweeteners | 31 (0-31) | 120 | |  | |
|  |  |  |  | |  | |
|  | For an individual, an optimal energy intake to maintain a healthy weight will depend on body size and level of physical activity. Processing of foods such as partial hydrogenation of oils, refining of grains, and addition of salt and preservatives can substantially affect health but is not addressed in this table. Wheat, rice, dry beans, and lentils are dry, raw. †Mix and amount of grains can vary to maintain isocaloric intake. ‡Beef and lamb are exchangeable with pork and vice versa. Chicken and other poultry is exchangeable with eggs, fish, or plant protein sources. Legumes, peanuts, tree nuts, seeds, and soy are interchangeable. §Seafood consist of fish and shellfish (eg, mussels and shrimps) and originate from both capture and from farming. Although seafood is a highly diverse group that contains both animals and plants, the focus of this report is solely on animals.  ¶ Unsaturated oils are 20% each of olive, soybean, rapeseed, sunflower, and peanut oil.  ║ Some lard or tallow are optional in instances when pigs or cattle are consumed. | | |  | |  |

Supplemental table 2: Description of included food components for the components in the EAT-Lancet diet index.

| **Components in the EAT-Lancet diet index** | |
| --- | --- |
| 1.Whole grains | Millets, wheat, maize, sorghum. The foods that are made from refined grains, such as maize ugali, chapati, white bread, and Mandazi, were excluded. |
| 2. Root and tubers | Boiled potatoes, fried potatoes, deep fried potatoes, boiled sweet potatoes, fried sweet potatoes, boiled cassava, boiled taro, boiled beetroot, boiled green banana, and boiled yams. |
| 3. Vegetables | All vegetables except legumes. |
| 4. Fruits | Fruits and berries. |
| 5. Dairy | Whole milk or derivative equivalents. Regular milk, low-fat milk, yoghurt and other fermented milk products, hard cheese, soft cheese, cream, butter, butter-based spreads. In the EAT-Lancet diet all dairy foods are expressed as of milk equivalents. The milk equivalents we used are based on the approach used by Stockholm Resilience Centre, based on ‘total solids’ and intakes of different dairy products were consequently multiplied with the following factors; whole milk 1.0, Cheese 5.0, cream 2.7 and butter 6.5 [2]. |
| 6. Beef and lamb | Beef, lamb, minced meat with pork and lamb, processed meats with beef and lamb including sausages. |
| 7. Pork | Pork, minced meat of pork, processed meats with pork including ham, bacon, and sausages. |
| 8. Chicken | Chicken, turkey, duck, goose, and other poultry. |
| 9. Eggs | Boiled eggs, fried eggs and eggs in dishes such as omelet and pie. |
| 10. Fish | Fatty fish, lean fish, fish products, shellfish. |
| 11. Legumes | Dry beans, lentils, peas, soy. Targets and index refer to raw weight. Peas, lentils, beans, tofu, soy containing meat replacement products. |
| 12. Nuts | Peanuts or tree nuts. All nuts and seeds including peanuts, nut mixes such as almond paste. |
| 13. Unsaturated oils | All plant oils and plant margarines. |
| 14. Added sugar | Sucrose and monosaccharides except sugars in fruits and vegetables [3]. |

**Supplemental table 3.** Criteria for the EAT-Lancet index developed by Stubbendorff et al [4]. All amounts are dry (uncooked) weight.

| **Food components in EAT-Lancet diet index (g/day)** | | **Target intake (reference interval)^2^** | **3 pts** | **2 pts** | **1 pt** | **0 pts** | **Criteria for score distribution** |
| --- | --- | --- | --- | --- | --- | --- | --- |
| **Emphasized intake** | Vegetables | 300 (200-600) | >300 | 200-300 | 100-200 | <100 | **Positive score**  3 pts = intake above target intake  2 pts = lower limit of reference interval up to target intake  1 pt = 50-100% of lower limit of reference interval  0 pts = < 50% of lower limit of reference interval |
|  | Fruits | 200 (100-300) | >200 | 100-200 | 50-100 | <50 |  |
|  | Oils | 40 (20-80) | >40 | 20-40 | 10-20 | <10 |  |
|  | Legumes | 75 (0-150) | >75 | 37.5-75 | 18.75-37.5 | <18.75 | **Positive score, adjusted^3^**  3 pts: intake above target intake  2 pts: 50-100% of target intake  1 pt: 25-50% target intake  0 pts: 0-25% of target intake |
|  | Nuts | 50 (0-100) | >50 | 25-50 | 12.5-25 | <12.5 |  |
|  | Whole grains | 232 | >232 | 116-232 | 58-116 | <58 |  |
|  | Fish | 28 (0-100) | >28 | 14-28 | 7-14 | <7 |  |
| **Limited intake** | Beef and lamb | 7 (0-14) | <7 | 7-14 | 14-28 | >28 | **Inverse score**  3 pts: intake below target intake  2 pts: target intake to upper limit of reference interval  1 pt: 100-200% of upper limit of reference interval  0 pts: > 200% of upper limit of reference interval |
|  | Pork | 7 (0-14) | <7 | 7-14 | 14-28 | >28 |  |
|  | Poultry | 29 (0-58) | <29 | 29-58 | 58-116 | >116 |  |
|  | Eggs | 13 (0-25) | <13 | 13-25 | 25-50 | >50 |  |
|  | Dairy | 250 (0-500) | <250 | 250-500 | 500-1000 | >1000 |  |
|  | Potatoes | 50 (0-100) | <50 | 50-100 | 100-200 | >200 |  |
|  | Added sugar | 31 (0-31) | <31 | 31-62 | 62-124 | >124 |  |

Tertile -2

N = 125

Tertile-3

N = 109

Tertile -1

N = 100

Older adults who consented to participated in the study from the 551 eligible older adults in 417 HHs

N = 417 older adults (one older adult per HH)

Households (HHs) with older adults (aged ≥ 55 years) listed from the 31 villages

N = 1351 HHs

Thirty-one villages sampled from 15 sectors of Gasabo district

Households declined to participate

N = 3 HHs

Sampled HHs with older adults

N = 420 HHs

HHs accepted to participate in the study

N = 417 HHs, with 551 older adults

Participants who participated in dietary assessment (24-h recall)

N = 401

Participants with incomplete data on two-days dietary (24-h) recalls

N = 7

Participants who provided two complete 24-h recall

N = 394

Lost to follow up (N = 16)

- 1 dead
- 2 relocated
- 1 hospitalized
- 1 voluntary withdraw
- 11 unavailable for further participation

Participants with extremely low (<600 Kcal) or high (>4200 Kcal) energy intake

N = 60

Included in the analysis

N = 334

**Supplementary Figure 1**: Flowchart for the selection of the participants

**References**

1. Willett, W., et al., *Food in the Anthropocene: the EAT-Lancet Commission on healthy diets from sustainable food systems.* Lancet, 2019.

2. Stockholm Resilience Centre, *Nordic food systems for improved health and sustainability. Baseline assessment to inform transformation*. 2019.

3. Ramne, S., et al., *Association between added sugar intake and mortality is nonlinear and dependent on sugar source in 2 Swedish population-based prospective cohorts.* Am J Clin Nutr, 2019. **109**(2): p. 411-423.

4. Stubbendorff, A., et al., *Development of an EAT-Lancet index and its relation to mortality in a Swedish population.* The American Journal of Clinical Nutrition, 2021.
